# Regulatory Consolidation to Contractual Architecture: The UAE's Emerging Takaful Framework

**DOI:** 10.12688/f1000research.190263.1

**Published:** 2026-09-22

**Authors:** Moatasem el-Gheriani, Alia Husam, Mohamad Hidayat Muhtar

**Affiliations:** 1 Private Law, United Arab Emirates University College of Law, AlAin, United Arab Emirates

**Keywords:** Takafu, Islamic Finance, Sharia Standards, Financial Regulation, UAE Law

## Abstract

**Purpose:**

This article evaluates whether the United Arab Emirates’ recent reforms have converted takaful from a specialist insurance arrangement supported mainly by regulatory and contractual instruments into a coherent statutory and regulatory architecture.

**Design/methodology/approach:**

The article uses doctrinal legal research. It reads the official Arabic legislation as authoritative and uses official English translations and the Central Bank of the UAE (CBUAE) Rulebook for accessibility. The analysis compares the 2010 takaful regulations, the 2018–2023 reforms, Federal Decree-Law No. 6 of 2025, the current CBUAE Regulation Regarding Takaful Insurance, and Article 967 of Federal Decree-Law No. 25 of 2025. Legal certainty is assessed across five distinct dimensions: the regulatory benchmark for Shari’ah compliance, institutional authority, fund and asset segregation, contractual characterization and remedies, and cross-border choice of law.

**Findings:**

The reforms materially strengthen regulatory, institutional and asset-segregation certainty. Federal Decree-Law No. 6 of 2025 maintains a state-backed Shari’ah governance hierarchy, requires the takaful fund to have legal personality and a financial liability distinct from the operator, and authorizes detailed CBUAE implementation. Article 967 supplies a civil-law point of recognition for the mutual takaful arrangement. The current CBUAE regulation further separates the fund’s substantive liability from the operator’s procedural and managerial accountability by making the company the sole suable party under the policy while requiring payment from fund assets. These developments do not, however, establish that every Shari’ah breach automatically invalidates a contract, nor do they guarantee that a foreign court will give effect to a UAE governing-law clause without applying its own conflict rules and mandatory insurance law.

**Practical implications:**

Operators should align the takaful policy, Participation Membership Policy, fund charter, Wakala terms and governing-law clause. Cross-border documentation should choose UAE law expressly, identify the relevant CBUAE and Higher Shari’ah Authority standards, and select a forum capable of applying that law. The reforms reduce important categories of uncertainty, but careful drafting remains necessary.

**Originality/value:**

The article offers an integrated doctrinal account of the 2025–2026 statutory reforms and their implementing regulatory architecture. It demonstrates that the UAE model improves legal certainty not through nomination alone, but through the interaction of public supervision, a legally distinct fund, a defined agency model and civil-law recognition.

## 1. Introduction

On 1 June 2026, Federal Decree-Law No. 25 of 2025 promulgating the UAE Civil Transactions Law entered into force. Article 967 now recognizes mutual takaful insurance in the Code’s chapter on insurance. That provision followed two other major developments: the regulation of Islamic financial transactions in Federal Decree-Law No. 50 of 2022 on Commercial Transactions, and the consolidation of central banking, financial regulation and insurance supervision in Federal Decree-Law No. 6 of 2025. Read together with the current CBUAE Regulation Regarding Takaful Insurance, these measures place takaful within a more integrated legal structure than the one considered in earlier scholarship on UAE Islamic insurance ( Ercanbrack, 2019; Jadalhaq and Russi, 2020).

Recent scholarship has examined the financial performance, governance attributes and dispute-resolution environment of Islamic financial institutions, including takaful ( Aljughaiman *et al.*, 2024; Bantekas, 2021; BenSaid, 2025; Brody, 2022). The UAE-specific legal literature most directly concerned with takaful, however, predates the 2025 Central Bank Law, the current CBUAE takaful regulation and the 2026 commencement of the new Civil Transactions Law ( Ercanbrack, 2019; Jadalhaq and Russi, 2020). The resulting gap is temporal and doctrinal: the new instruments have not yet been analysed as a single architecture linking public Shari’ah supervision, fund personality, operator accountability, nominated-contract recognition and governing law.

The central question is not whether the new framework uses the language of legal certainty. It is whether the framework changes the legal position of the institutions, assets and contracts involved in a takaful operation. That question requires a narrower inquiry than the claim that codification necessarily makes the UAE the preferred law for all Islamic finance transactions. It also requires more than a description of the relevant provisions. The legal effect of a takaful reform depends on how the rules allocate authority between the CBUAE, the Higher Shari’ah Authority (HSA), the operator and its Internal Shari’ah Supervision Committee (ISSC); how they allocate property and liability between the operator and the fund; and how they connect regulatory compliance with contractual rights and judicial remedies.

This article argues that the reforms create a layered and increasingly coherent architecture. The 2025 Central Bank Law centralizes the public-law benchmark for Shari’ah compliance and restates the institutional hierarchy through which that benchmark is implemented. It also develops the legal personality and independent financial liability of the takaful fund. The current CBUAE regulation supplies the operational bridge between the fund and the company by imposing a Wakala model, segregating accounts, and making the company accountable as the sole suable party even though policy obligations are discharged from fund assets. Article 967 then gives the basic mutual arrangement an express civil-law basis. The combined effect is substantial improvement in regulatory, institutional and asset-segregation certainty.

The article does not claim that the reforms eliminate every private-law or cross-border issue. Neither the Central Bank Law nor the current takaful regulation expressly provides that every departure from an HSA or ISSC standard renders the relevant policy void or unenforceable. Article 967 identifies the mutual structure but does not itself provide a complete code for ownership, insolvency, agency, surplus, deficit or remedies. A UAE governing-law clause is also distinct from a general contractual reference to Shari’ah, yet its effectiveness abroad remains subject to the forum’s conflict rules, mandatory law and rules on proof of foreign law. These qualifications do not diminish the reform. They identify what the reform has accomplished and what must still be supplied by regulation, contract or adjudication.

The article makes three contributions. First, it reconstructs the reform sequence rather than treating Federal Decree-Law No. 6 of 2025 and Article 967 as isolated enactments. This reveals both continuity and change: for example, the repealed Federal Decree-Law No. 48 of 2023 already recognized the fund’s independent legal personality, while the 2025 law adds distinct financial liability, registration, a separate constitutional document and financial disclosure. Second, it separates five meanings of legal certainty that are frequently collapsed into a single claim. Third, it connects the public-law governance system to the private-law structure of the policy and to cross-border governing-law analysis.

The discussion proceeds as follows. Section 2 explains the doctrinal method and analytical framework. Section 3 identifies the legal relationships that make takaful distinct and explains why the market requires a clear allocation of authority and assets. Section 4 examines the consolidation of Shari’ah governance. Section 5 analyses the legal personality of the fund and the operator’s accountability. Section 6 evaluates Article 967 as nominated-contract recognition. Section 7 considers governing law and cross-border enforcement. Section 8 identifies implementation priorities, and Section 9 concludes.

## 2. Research methodology

This is a doctrinal study of UAE federal law as at 4 August 2026. Its primary materials are the official Arabic texts of the relevant federal decree-laws, the official English translations published by the UAE Government, and CBUAE regulations and standards. The Arabic text remains authoritative where an official translation differs from it. The study therefore treats translated terminology such as “legal personality”, “financial liability”, “contribution”, “fund” and “Wakala” as legal terms whose effect must be established from the legislative structure, not from an English label in isolation.

The analysis is chronological and functional. Chronologically, it compares the Insurance Authority’s 2010 Takaful Insurance Regulations; the creation and development of the HSA under the 2018 Central Bank legislation; the transfer of insurance supervision to the CBUAE under Federal Decree-Law No. 24 of 2020; the Commercial Transactions Law of 2022; the insurance legislation of 2023; and the 2025–2026 reforms. Functionally, it asks what each instrument contributes to the regulation of the Shari’ah benchmark, the authority of institutions, the identity and assets of the fund, the contractual relationship, and dispute resolution.

Legal certainty is used here in five separate senses. Regulatory certainty concerns the standard by which a licensed institution is treated as Shari’ah-compliant. Institutional certainty concerns which public and private bodies may make, supervise and resolve Shari’ah determinations. Asset and liability certainty concerns who owns, manages and may answer for the contributions and other assets allocated to the fund. Contractual certainty concerns the characterization of the takaful arrangement, the parties’ rights, and the consequences of breach. Cross-border certainty concerns whether parties can select UAE law and obtain recognition of that selection before a foreign court or tribunal. A reform can improve one dimension without resolving another.

Four distinctions follow from this framework. First, regulatory non-compliance is not automatically equivalent to civil invalidity. A statute may authorize supervisory sanctions without creating a private cause of action or a rule of nullity. Second, nomination of a contract is not the same as comprehensive codification. A named provision may supply legal recognition while leaving many incidents to special law and contract. Third, separate legal personality does not by itself answer every question of standing, representation, insolvency priority or creditor access. Fourth, choosing a state law that incorporates Shari’ah standards is different from selecting “Shari’ah” as a free-standing non-state governing law. These distinctions organize the argument and prevent claims of certainty from exceeding the legal materials.

The article is not an empirical assessment of the economic effects of the reform. The Civil Transactions Law has been in force only since 1 June 2026, and the present regulatory architecture has not yet generated a body of reported judicial decisions sufficient to measure litigation outcomes. Statements about transaction costs, consumer confidence or capital attraction are therefore framed as plausible implications, not demonstrated findings. The article’s claim is doctrinal: the reforms alter the legal architecture in ways that can reduce specified forms of uncertainty.

## 3. The legal architecture and market relevance of takaful

Takaful is not defined merely by the absence of interest or by a label of Shari’ah compliance. Under Article 967 of the Civil Transactions Law and the current CBUAE regulation, participants make monetary contributions to a fund for mutual protection against specified risks. The contribution is paid on the basis of tabarru’, and the fund is responsible for compensation or benefits when an insured risk materializes. The company does not take the contribution as its own premium in exchange for assuming the risk on its balance sheet. It manages the fund and related insurance operations as an agent under a Wakala structure prescribed by the current regulation.

The legal relationships differ according to the business line. For property and liability takaful, contributions, investment returns, and retakaful revenues are allocated to the fund, and claims are paid from its assets. For insurance of persons and fund accumulation, the current regulation separates the risk-coverage portion paid to the fund from the investment portion placed in a Participants’ Investment Account and managed under Wakala bi al-istithmar. The Shareholders’ Account represents the assets and liabilities of the company. The legal architecture must therefore distinguish at least the participant, any designated beneficiary, the fund, the operator, the shareholders’ account and, in family takaful, the investment account.

This structure explains why a general statement that participants are “co-insurers” is inadequate. Participants contribute to mutual protection, but the current regulation centralizes claims administration and external accountability in the licensed company. The fund owns the donated contributions and bears the policy liability from its assets, while the company represents and manages the fund. The model combines mutuality, a distinct asset pool and professional intermediation. Its legal coherence depends on the alignment of several documents: the takaful policy, the Participation Membership Policy, the fund charter, the Wakala arrangement, the surplus policy, the deficit policy and the applicable Shari’ah standards.

The sector is economically smaller than Islamic banking but legally important to the wider Islamic finance ecosystem. The CBUAE’s 2024 insurance statistics reported ten national takaful companies and national takaful gross written contributions of approximately AED 5.48 billion. The same reporting placed takaful at approximately 8.4 per cent of total UAE insurance-sector gross written premiums when foreign branches were included ( Central Bank of the UAE, 2024). Internationally, the Islamic Financial Services Board reported continued growth in takaful assets and contributions, with the Gulf Cooperation Council remaining the largest regional market ( Islamic Financial Services Board, 2025). These figures provide context, but they do not prove that the 2025–2026 reforms have caused market growth.

Before the latest reforms, three legal problems recurred. The first was dispersion of the Shari’ah benchmark across international standards, regulator decisions and institution-level opinions. The second was the need to separate participant assets from shareholder assets while preserving an administratively workable claims process. The third was the weak connection between a regulatory finding of Shari’ah compliance and the private-law characterization and cross-border enforcement of a transaction. Earlier UAE regulation addressed important parts of these problems, but the reforms now connect them more expressly through legislation, regulatory standards and civil-law recognition.

## 4. Centralized Shari’ah governance and the scope of regulatory certainty

### 4.1 Legislative consolidation and transition

The present framework is evolutionary rather than a complete break with the past. The Insurance Authority’s Board Resolution No. 4 of 2010 established a detailed sector-specific regime. Federal Decree-Law No. 14 of 2018 placed the HSA within the central banking framework, and HSA Resolution No. 18/3/2018 adopted AAOIFI Shari’ah standards as minimum requirements for ISSCs from 1 September 2018. ( AAOIFI, 2017; CBUAE Rulebook, 2020) Federal Decree-Law No. 24 of 2020 then transferred insurance supervision to the CBUAE. These measures created the institutional foundations on which the later reforms were built.

Federal Decree-Law No. 50 of 2022 on Commercial Transactions added a statutory layer. ( UAE Legislations, 2022) Its provisions on Islamic financial institutions require compliance with the Shari’ah criteria and controls issued by the HSA and authorize the CBUAE to regulate the commercial transactions of Islamic financial institutions and takaful companies. The Commercial Transactions Law also lists takaful among the Islamic financial transactions within its regulatory chapter, but it does not provide the civil definition later supplied by Article 967. Its principal contribution is therefore the recognition of HSA-based standards within the law governing commercial activity rather than a complete takaful contract code ( El-Gheriani, 2025).

Federal Decree-Law No. 48 of 2023 was an important intermediate step. Articles 69–73 created a dedicated takaful chapter, and Article 70 required a fund with independent legal personality. Contributions, investment returns and retakaful revenues were allocated to that fund, which bore operational expenses and policy compensation. The 2025 reform should not therefore be described as introducing fund separation for the first time. Its contribution lies in consolidation and elaboration. ( UAE Legislation, 2023).

Federal Decree-Law No. 6 of 2025 repealed both the 2018 Central Bank legislation and Federal Decree-Law No. 48 of 2023, while combining central banking, regulation of financial institutions and insurance business in one statute. ( CBUAE Rulebook, 2025) Article 183 preserves existing regulations, standards, decisions, guidelines and circulars until they are replaced, and Article 184 grants regulated persons a period to reconcile their position with the new law. This transition mechanism prevents a regulatory vacuum, but it also means that the framework must be read as a combination of new legislation and continuing subordinate instruments. Legal certainty during transition depends on identifying which instruments remain in force and which have been superseded.

The current CBUAE Regulation Regarding Takaful Insurance now expressly supersedes the 2022 regulation. It must be distinguished from two further standards displayed by the CBUAE with an effective date of 15 July 2027: the Standard Re the Operational Model of Takaful Insurance and the Standard Re Controls and Procedures Establishing Takaful Insurance Fund. ( CBUAE Rulebook, 2025, 2026) Those standards are important evidence of the direction of implementation, but their operative requirements should not be represented as already applicable before their stated effective date. The reform is therefore best understood as a current statutory and regulatory core accompanied by a scheduled next stage of operational specification.

### 4.2 The public benchmark and institutional hierarchy

Article 24 of the 2025 Central Bank Law maintains the HSA as the central public reference for the Shari’ah rules, standards and principles governing Islamic financial institutions. Article 74 connects the licensing regime to that authority: an Islamic financial institution is treated as compliant when it complies with HSA resolutions, regulations and standards. This converts an otherwise open-ended reference to Shari’ah into a regulator-administered benchmark for licensed activity. The legal force of an AAOIFI standard in this setting does not arise merely because AAOIFI is an influential non-state standard setter. It arises because the HSA has adopted the standard within the statutory regulatory hierarchy.

This distinction answers part of the concern that future amendments by a private standard setter could automatically alter UAE law. HSA Resolution No. 18/3/2018 adopted existing and future AAOIFI standards as minimum requirements for ISSCs. Yet an institution’s public-law duty remains mediated by the HSA and the CBUAE framework. The relevant legal question is not whether AAOIFI can legislate for the UAE; it cannot. The question is how the UAE regulator has incorporated, qualified or supplemented AAOIFI standards through an authority created by federal law. Dynamic incorporation may still require transparency and careful identification of the applicable version, but it does not eliminate the state-law source of the obligation.

Article 75 requires each Islamic financial institution, including a takaful company, to establish an independent ISSC. The committee supervises products, services, contracts, documents and conduct, and its resolutions bind the institution when consistent with HSA resolutions. The statute also requires independent internal Shari’ah control and internal Shari’ah audit functions. The CBUAE’s 2023 Shari’ah Governance Standard for Takaful Insurance Companies organizes these functions through a three-lines-of-defence model and leaves ultimate responsibility with the board. ( CBUAE Rulebook, 2023) The result is not private self-certification. It is supervised internal governance within a public regulatory hierarchy.

The allocation of functions is significant. The HSA supplies the general benchmark and resolves specified institutional disagreements. The ISSC applies that benchmark to the company’s products and operations. Management implements the approved structure. Internal Shari’ah control monitors first-line execution, internal Shari’ah audit provides independent assurance, and external Shari’ah audit may add further review. The CBUAE retains licensing, supervision and enforcement powers. Recent research on Islamic finance governance supports the proposition that Shari’ah boards operate as an additional governance layer, but the UAE framework goes further by embedding that layer in state supervision rather than relying on board composition alone ( Aljughaiman *et al.*, 2024).

The current takaful regulation applies this hierarchy to the fund and the policy. It requires the company to comply with Shari’ah provisions in its products, operations, policies, charters and codes of conduct. ISSC resolutions bind the company if they do not conflict with HSA decisions. The regulation also requires separate internal Shari’ah control and audit departments and an annual Shari’ah report. These mechanisms improve regulatory certainty because they identify both the applicable benchmark and the bodies responsible for translating that benchmark into operational controls.

### 4.3 Regulatory breach and civil effect

The framework is clearest about regulatory consequences. Article 77 of the 2025 Central Bank Law authorizes the CBUAE, after consultation with the HSA, to take action when an institution contravenes Shari’ah provisions as defined by HSA rulings, resolutions and standards. The current takaful regulation likewise subjects proven non-compliant activity to CBUAE enforcement, and its general enforcement provision permits supervisory measures and sanctions. These rules make Shari’ah compliance a public-law obligation rather than a matter of marketing discretion.

The private-law consequence is less explicit. The legislation does not state that every contravention automatically voids the relevant takaful policy, removes coverage, or creates a direct damages action for a participant. Nor would automatic nullity necessarily protect participants: invalidating a policy after a loss could transfer the cost of the operator’s regulatory breach to the person the regulatory system is intended to protect. A court would need to consider the statutory purpose, the mandatory character of the relevant rule, the policy wording, causation, and the remedies available under the Civil Transactions Law and special insurance legislation.

The defensible conclusion is therefore limited but important. HSA and CBUAE standards provide an authoritative benchmark for regulatory compliance and can inform the interpretation of the policy, the fund charter and the operator’s contractual duties. A specific standard incorporated into the documentation may also operate as an express contractual term. Yet regulatory bindingness alone does not establish a uniform rule of civil invalidity. Future case law or legislation may clarify whether particular breaches produce nullity, damages, restitution, regulatory remediation, or no private remedy beyond enforcement by the CBUAE.

This distinction should shape drafting. A policy should not rely on a general warranty that the transaction is “Shari’ah-compliant” without identifying who determines compliance and what remedy follows from a breach. The documents should state whether identified HSA and CBUAE standards are incorporated as terms, whether a later change applies to existing policies, how an ISSC determination affects the parties, and whether non-compliance requires correction, compensation or another remedy. The state-backed benchmark reduces interpretive uncertainty, but contractual precision determines how that benchmark operates between the parties.

## 5. The takaful fund: legal personality, asset segregation and operator accountability

### 5.1 Continuity and development from the 2023 law

The fund is the point at which the reform most clearly changes legal architecture. Article 70 of Federal Decree-Law No. 48 of 2023 already required a takaful fund with independent legal personality. ( UAE Legislation, 2023) The fund received contributions, investment returns and retakaful revenues, paid insurance expenses and bore policy compensation. Federal Decree-Law No. 6 of 2025 retains that premise but makes the separation more explicit. ( CBUAE Rulebook, 2025) Article 105 requires the fund to have both legal personality and a financial liability distinct from the company, to be registered with the CBUAE, to have its own articles of association, and to disclose an independent financial position in the company’s financial statements.

The current regulation develops these elements. It defines the fund as owning the paid contributions donated by participants. It requires a separate fund for insurance of persons and fund accumulation and for property and liability business. Article 18 requires complete separation of the fund’s assets and liabilities from those of the company, while separately identifying the Participants’ Investment Account and Shareholders’ Account. The distinction is therefore proprietary and accounting-based, not merely a bookkeeping convention within a single corporate estate.

Separate personality performs three functions. First, it identifies an asset pool to which contributions and specified revenues belong. Second, it identifies the source from which compensation and benefits are to be discharged. Third, it limits the operator’s role to management and representation rather than beneficial ownership of the fund. These functions support participant protection by reducing the legal and operational risk of commingling participant assets with shareholder assets.

The reform should nevertheless not be described as establishing a complete statutory trust or a fully specified mutual company. Article 105 does not classify participants as members with corporate voting rights, creditors, beneficiaries of a trust, or owners of aliquot shares in fund assets. The current regulation describes the fund as owner of the contributions and the participant as a beneficiary of the fund, subject to the policy and Participation Membership Policy. That is a more precise allocation than the former language of a participant’s account, but questions may remain concerning residual assets on liquidation, creditor priority, and the treatment of undistributed surplus.

Nor does independent financial liability alone establish insolvency remoteness in every circumstance. The assets and liabilities are expressly separated, and the company does not own the contributions. Those rules provide a strong basis against ordinary commingling. However, the priority of participant claims in an insolvency, the effect of avoidance rules, the treatment of common expenses, and the interaction with the resolution powers in the Central Bank Law require the application of the relevant insolvency and insurance provisions. It is more accurate to say that the reform strengthens ring-fencing than to claim, without further authority, that the fund is immune from every creditor claim.

### 5.2 Substantive liability and procedural standing

The current regulation resolves a practical difficulty that can arise from separate fund personality. Article 6 provides that all beneficiary entitlements under a takaful policy are enforceable solely against the company, which is the sole suable party. At the same time, the fund is responsible for the policy obligations, payment is made directly from fund assets managed by the company, decisions of the company on claims bind the fund, and the company remains fully accountable for proper and timely settlement. The structure separates the source of payment from the procedural respondent.

This is not a contradiction. The fund’s independent personality and liability protect the asset pool and identify the substantive account on which claims fall. The sole-suable-party rule gives participants and beneficiaries a licensed and supervised defendant instead of requiring them to determine whether to sue the fund, its trustees, the operator in its own capacity, or several parties. The company is answerable because it issued and administers the policy as representative of the fund. The fund remains economically liable because the obligation is discharged from its assets.

The rule also clarifies the significance of agency. The regulation defines the takaful policy as a contract between the company, acting as representative of the fund, and the participant. It defines the Wakala contract as the fund’s appointment of the company to manage the fund for a fee, and, where applicable, the participant’s appointment of the company to manage the investment account. Agency is therefore not an explanatory metaphor. It is the legal mechanism connecting a separate fund to a professional operator and to third-party claimants.

Agency does not eliminate conflicts of interest. The operator sets fees, manages claims, arranges retakaful, invests assets and may receive a permitted share of surplus for sound management. The current regulation responds through board responsibility, ISSC oversight, actuarial approval, CBUAE approval of surplus distributions, separate accounts and disclosure. A fund Board of Trustees may be established, and the CBUAE may require one. The standards scheduled for 15 July 2027 further specify the fund charter, representation and trustee arrangements. These mechanisms should be assessed as controls on the operator’s agency discretion, not as substitutes for it.

### 5.3 Deficit, qard hasan and solvency

The treatment of deficit shows why regulatory and contractual obligations must be separated. Article 21 of the current regulation requires the company to provide qard hasan when fund assets are insufficient to meet liabilities, up to the stated regulatory limit. The regulation expressly provides that this obligation is not a contractual commitment to the fund; it exists to comply with the regulation. The company may recover the qard from future surplus with the required approvals.

This design protects current claims without converting the operator into the primary risk bearer in ordinary circumstances. The fund remains liable for policy obligations, but the operator must supply regulatory support when the fund is deficient. Describing the qard as non-contractual also limits the inference that participants may sue for the loan as a promised policy benefit. The CBUAE can enforce the obligation, including through supervisory measures if the company fails to provide the required support.

Solvency is assessed on a consolidated basis at company level under the current regulation, while the fund must maintain and disclose a separate financial position. Where a fund is in recurring deficit or relies on qard, the CBUAE may require an independent fund-level solvency assessment. This approach recognizes economic interdependence without dissolving legal separation. It also prevents a healthy shareholder account from obscuring persistent weakness in the fund.

The framework materially improves asset and liability certainty, but implementation remains important. The fund charter and Participation Membership Policy should state the allocation of contributions, expenses, investment income, retakaful proceeds, surplus, and qard recovery. Financial statements should permit participants to identify the fund’s position without conflating it with shareholder performance. The scheduled 2027 standards can further reduce ambiguity by standardizing the contractual map and legal representation of the fund.

## 6. Article 967 and the value of nominated-contract recognition

Article 967 of the Civil Transactions Law provides that several persons may conduct mutual takaful insurance through monetary contributions intended to achieve solidarity and cooperation against specified risks; each contribution is deposited in a takaful fund in accordance with applicable legislation; every member of the system is insured on a takaful basis; and the funds may be invested with returns distributed according to agreement. The provision identifies mutuality, contribution, the fund, risk protection and possible investment as the basic elements of the arrangement.

The provision should be read with the surrounding insurance chapter and with special legislation. Article 953 makes the insurance chapter subject to applicable special laws, while Article 955 supplies the general definition of insurance. The Civil Transactions Law also preserves the principle that a specific rule governs within its field where it conflicts with a general rule. Article 967 therefore does not displace the Central Bank Law or CBUAE regulation. It supplies the civil-law point of entry through which the court identifies the arrangement; special law supplies the institutional and prudential architecture.

This interaction is the principal value of nomination. Before Article 967, a court could still enforce a takaful arrangement under general contract rules, special insurance legislation and the parties’ documents. The absence of a named provision did not mean that the contract was legally nonexistent. Nomination nevertheless reduces characterization risk. A court no longer needs to decide at the threshold whether the arrangement is an unnamed combination of donation, agency, investment and insurance. It can begin with a statutory category that recognizes the mutual structure and directs the arrangement toward applicable legislation.

The nomination also disciplines contractual interpretation. The use of “contribution” and “takaful insurance fund” supports the separation of participant assets from the operator. The recognition that each member is insured on a takaful basis prevents the donation element from being used to deny the protective purpose of the transaction. The possibility of investment, coupled with distribution according to agreement, confirms that investment rights require contractual specification rather than arising automatically from the risk contribution.

Article 967 does not, by itself, answer who owns the fund, who represents it, who may be sued, how fees are calculated, how surplus is distributed, who finances a deficit, or what happens on insolvency. Those questions are answered, to varying degrees, by Article 105 of the Central Bank Law, the current takaful regulation, the policy and related documents. It is therefore inaccurate to attribute the fund’s full architecture to Article 967 alone. The Code recognizes the type; the regulatory system constructs the institution.

The same point applies to remedies. Article 967 does not create a special remedy for failure to comply with an HSA standard, nor does it state that a policy is void if the operator departs from the approved model. General contract and insurance remedies remain relevant, subject to mandatory special legislation. Nomination improves the legal basis for pleading and interpretation, but it does not replace the need to identify the breached obligation and the remedy attached to it.

The provision’s brevity may be an advantage. A detailed civil code model could become obsolete as actuarial practice, product structures and CBUAE standards evolve. Article 967 instead states the stable private-law core while referring to applicable legislation. The trade-off is that certainty depends on coordination between the Code, the Central Bank Law and subordinate instruments. The UAE model is thus better described as coordinated codification than as exhaustive codification.

## 7. Choice of law and cross-border effect

### 7.1 From a reference to Shari’ah to a choice of UAE law

The cross-border argument must distinguish a state-law clause from a non-state-law clause. In Beximco Pharmaceuticals Ltd v Shamil Bank of Bahrain EC [2004] EWCA Civ 19, the Court of Appeal treated English law as the governing law and declined to give an additional general reference to Shari’ah the effect of a second governing law. ( Casemine, 2004) The decision has generated substantial criticism, particularly concerning party autonomy and the possibility of incorporating sufficiently specific non-state rules ( Bantekas, 2021; Brody, 2022). It nevertheless illustrates the drafting risk of combining a state law with an unidentified body of Shari’ah principles.

A clause choosing UAE law after the current reforms is legally different. It selects the law of a state. That state law includes the Civil Transactions Law, the Central Bank Law, applicable CBUAE regulations, and the legally relevant effect of HSA standards. The parties are not asking a foreign court to identify Shari’ah as an autonomous system without a national conflict-of-laws anchor. They are selecting a national law that has incorporated a regulatory benchmark for Shari’ah-compliant financial activity.

Article 19 of the 2025 Civil Transactions Law reinforces this position within UAE private international law by recognizing an express party choice for contractual obligations. ( UAE Legislations, 2025) Where UAE law is chosen, and the dispute is heard in an onshore UAE court, Article 967 and the special regulatory framework provide an identifiable body of state law. This is a stronger legal basis than a clause stating that a contract is governed by another national law “subject to Shari’ah” without identifying the relevant standards or their contractual effect.

The improvement should not be overstated. Article 19 governs the approach of UAE courts; it does not bind a foreign court. A foreign forum will apply its own conflict rules to determine whether the choice is valid, whether mandatory insurance law overrides it, and how UAE law is proved. The applicable regime may also differ in the DIFC or ADGM, whose courts apply their own laws and conflict rules. “UAE law” should therefore be drafted precisely to identify onshore federal law where that is the intended choice.

### 7.2 Lessons from Beximco, Dana Gas and non-state standards

Beximco does not establish that specific Shari’ah standards can never operate contractually. The judgment addressed a general formulation and the difficulty of identifying the rules said to qualify English law. Parties remain able, subject to the governing law, to incorporate sufficiently identified rules as contractual terms. Contemporary scholarship on non-state rules and instruments such as the Uniform Customs and Practice for Documentary Credits demonstrates that incorporation, governing law and arbitral application are distinct techniques ( Adams, 2022).

The Dana Gas litigation illustrates another limit. The main English merits judgment enforced an English-law purchase undertaking notwithstanding arguments concerning the validity of the underlying Islamic finance structure under UAE law. The English court did not convert a dispute about the underlying Mudarabah into a general ruling on Shari’ah. It applied the governing law and the separable contractual obligation before it. Dana Gas PJSC v Dana Gas Sukuk Ltd [2017] EWHC 2928 (Comm) therefore supports careful analysis of each document, governing-law clause and obligation; it does not show that national codification is irrelevant. ( Minwer Al-Rimawi, 2021; Mr Justice Leggatt, 2018).

For takaful, the practical lesson is document-specific. A policy governed by UAE law may incorporate the Participation Membership Policy, the fund charter and identified HSA or CBUAE standards. The Wakala agreement may have a different set of parties from the insurance policy, and a Participants’ Investment Account may raise additional proprietary and investment questions. A cross-border dispute must identify which obligation is sued upon, which document contains it, and which law governs that document. A general statement that the entire arrangement is “subject to Shari’ah” is unlikely to perform that work.

The UAE reforms improve the quality of a governing-law choice because they make the selected state law more specific to takaful. Article 967 supplies classification; Article 105 and the current regulation allocate liability, agency and assets; HSA and CBUAE standards provide the regulatory benchmark. These elements can reduce the need for a foreign court to construct the arrangement solely from expert evidence on an uncodified concept. They may also make expert evidence more focused because the question becomes the content and effect of identified UAE legal instruments.

The reforms do not guarantee universal enforcement. A foreign court may apply local mandatory insurance regulation, consumer protection, public policy or rules on direct actions. It may characterize a particular issue as proprietary, corporate or insolvency-related rather than contractual. Recognition of a UAE judgment or award raises separate procedural questions. The appropriate claim is therefore that the reforms make UAE law a more credible and administrable choice for takaful transactions, not that they compel foreign courts to prefer it.

### 7.3 Drafting implications

A cross-border takaful clause should expressly identify the chosen law, the forum and the language of authoritative materials. It should avoid an undefined dual-law formula. Where compliance with specified HSA or CBUAE standards is intended to create contractual rights, those standards should be identified and their effect stated. The documents should address whether amendments apply prospectively, how an inconsistency between an ISSC determination and an HSA standard is resolved, and whether the remedy is correction, damages, termination or another agreed response.

The parties should also align dispute-resolution clauses across the policy, Participation Membership Policy, fund charter and investment documentation. Fragmented clauses can produce parallel proceedings or inconsistent governing laws. Arbitration may permit the parties to select non-state rules more flexibly than some national courts, but the tribunal still requires a precise mandate and an enforceable award. Bantekas (2021) is therefore right to emphasize the compatibility of Islamic finance disputes with mainstream commercial arbitration, while the UAE reforms improve the state-law content that a tribunal may be asked to apply.

## 8. Remaining implementation priorities

The first priority is publication and version control. The legal effect of the framework depends on knowing which HSA, CBUAE and AAOIFI standards apply at the relevant time. The CBUAE Rulebook is an important access point, but it states that formally issued instruments prevail if an inconsistency arises. Operators should maintain a verified register of applicable standards and record the version used when a product is approved.

The second priority is coordination of contractual documents. The current regulation assigns functions to the policy, the Participation Membership Policy and the fund’s governance instruments. Each document should identify its parties, subject matter, hierarchy and relationship with the others. A participant should be able to determine which amount is donated to the fund, which amount is invested, what fees are charged, how a claim is processed, how surplus may be distributed and how qard is recovered.

The third priority is clarification of private-law consequences. Regulatory sanctions are well established, but the legal effect of Shari’ah non-compliance on an existing policy remains capable of generating dispute. Guidance or future case law should distinguish breaches that require regulatory correction from breaches that affect contractual validity or damages. The analysis should give priority to participant protection and avoid remedies that would deprive an innocent beneficiary of coverage because of an operator’s compliance failure.

The fourth priority is fund-level governance and insolvency treatment. The scheduled 2027 standards will further specify the fund charter, trustee arrangements and legal representation. Additional clarity may still be required on residual assets, participant representation, creditor access, liquidation and transfer of a fund. These questions are not evidence that separate personality has failed. They are the ordinary consequences of creating a legal person whose relationship with its participants and operator must be fully articulated.

Finally, claims about economic impact should be tested rather than assumed. The reforms plausibly reduce legal due-diligence costs and improve the administrability of products, but those effects require evidence from policy drafting, licensing practice, disputes, consumer complaints and market behavior. A future empirical study could examine whether standardized documentation, fund disclosure and centralized Shari’ah supervision change transaction costs or participant confidence after a sufficient implementation period.

## 9. Conclusion

The UAE’s recent reforms do more than add takaful terminology to existing insurance law. They connect a state-backed Shari’ah benchmark, supervised internal governance, a legally and financially distinct fund, an agency-based operating model and express civil-law recognition. The framework is cumulative: the 2018–2023 measures created important foundations, Federal Decree-Law No. 6 of 2025 consolidated and elaborated them, the current CBUAE regulation supplied an operational allocation of liability and accountability, and Article 967 placed the mutual arrangement within the Civil Transactions Law. The strongest improvement concerns regulatory, institutional and asset-segregation certainty. A licensed institution can identify the public benchmark for Shari’ah compliance. The roles of the HSA, ISSC, management, control and audit are allocated within a supervisory hierarchy. Contributions belong to a separate fund, fund assets answer for policy obligations, and the company remains the sole suable and accountable representative. Article 967 reduces characterization risk by recognizing the mutual structure and directing it to applicable special legislation. Two limits remain. The framework does not expressly make every Shari’ah breach a ground of civil invalidity, and nominated-contract recognition does not itself answer all questions of remedies, insolvency or participant governance. In cross-border cases, choosing UAE law is now more administrable than a general reference to Shari’ah, but foreign courts and tribunals will still apply their own conflict and enforcement rules.

The defensible conclusion is therefore neither that the reform is merely symbolic nor that it has eliminated legal uncertainty. The UAE has constructed a coherent legal architecture capable of supporting sophisticated takaful activity. Its long-term authority will depend on disciplined implementation, aligned documentation and judicial clarification of the remaining private-law questions.

## Use of generative AI

Generative AI was used solely for grammar checking, language refinement, and copyediting to improve manuscript readability. All ideas, analyses, and content remain the sole responsibility of the authors.

## Data Availability

No data are associated with this article.
