# Supplementary material for: Regulatory Consolidation to Contractual Architecture: The UAE's Emerging Takaful Framework
Source: F1000Res. 2026 Sep 22;15:1557. [Version 1] doi: 10.12688/f1000research.190263.1 (PMC13752447; doi:10.12688/f1000research.190263.1)
Supplement: Peer review history

# Reviewer report: Reviewer response for version 1

**DOI:** 10.5256/f1000research.210004.r523911

**Published:** 2026-10-07

**Authors:** Ssonko Muhammedi

**Affiliations:** 1 Sultan Abdul Halim Mu'adzam Shah International Islamic University, Kedah, Malaysia

**Summary of the article**

 The article analyses the transformation of takaful in the UAE due to recent legislative reforms. It utilizes doctrinal legal research to evaluate 2010 regulations, reforms from 2018 to 2023, Federal Decree-Law No. 6 of 2025, current CBUAE Regulations, and Article 967 of Federal Decree-Law No. 25 of 2025. The study assesses legal certainty in five areas: Shari’ah compliance, institutional authority, fund and asset segregation, contractual characterization and remedies, and cross-border choice of law.

 The authors highlight improvements in regulatory and institutional stability due to recent reforms, specifically regarding asset segregation. Federal Decree-Law No. 6 of 2025 introduces a Shari’ah governance framework, confirms the legal status of takaful funds, and assigns execution responsibilities to the Central Bank of the UAE (CBUAE). It recognizes mutual takaful within civil law and mandates the separation of fund liabilities from operator obligations. However, the authors warn that not all Shari’ah violations void contracts and foreign courts may not uphold UAE governing-law clauses. The article emphasizes the practical implications for operators, including the necessity for improved document coordination, clearer legal ramifications, and enhanced fund-level governance.

 The article highlights a significant gap in UAE-specific legal literature regarding takaful, as existing resources are outdated compared to recent legal changes, including the 2025 Central Bank Law and 2026 Civil Transactions Law. It offers a comprehensive analysis of these new developments and their regulatory framework.

 Major point

**Strengths**

1. The article makes a timely and original contribution by addressing a significant gap in literature regarding UAE-specific legal issues related to takaful. It effectively situates itself within the context of the 2025 Central Bank Law, the CBUAE takaful regulations, and the upcoming 2026 Civil Transactions Law. The authors assert that their work offers an integrated doctrinal account of these recent statutory reforms, a claim that is well substantiated by their analysis.
2. The text presents a clear analytical framework that categorizes legal certainty into five distinct dimensions: regulatory, institutional, asset and liability, contractual, and cross-border. This differentiation avoids the simplification of legal certainty and allows for nuanced arguments about the outcomes of reforms. The authors emphasize that improvements in one dimension do not necessarily resolve issues in others, highlighting a crucial methodological insight.
3. Balanced conclusions acknowledge that the reforms do not address all private-law or cross-border issues and that Article 967 is not a complete code for various legal aspects. The authors state that the reform is neither purely symbolic nor entirely eliminating legal uncertainty, reflecting the evidence presented.
4. Practical implications of cross-border takaful documentation are crucial for practitioners. Key recommendations include aligning the takaful policy, Participation Membership Policy, fund charter, Wakala terms, and governing-law clause. It is emphasized that cross-border documentation should clearly identify the applicable law, forum, and language of authoritative materials, which is a practical and analyzed approach.
5. Engagement with comparative and international legal materials is exemplified in Beximco Pharmaceuticals Ltd v Shamil Bank of Bahrain EC and the Dana Gas litigation, providing a foundation for cross-border analysis. The authors argue for the preference of UAE law over a general Shari'ah reference due to its superior administrability. Furthermore, the analysis includes the compatibility of Islamic finance disputes with mainstream commercial arbitration, enhancing the discussion through Bantekas (2021).
6. Clear implementation priorities are outlined, focusing on four areas: publication and version control, coordination of contractual documents, clarification of private-law consequences, and fund-level governance and insolvency treatment. These serve as a roadmap for future research and regulatory development.

**Weaknesses**

1. Methodology could improve with more detailed information on the systematic selection and analysis of legal materials. The article, a doctrinal study of UAE federal law as of 4 August 2026, employs Arabic texts of federal decree-laws, official English translations, and CBUAE regulations. However, it lacks clarity on the criteria for selecting these materials and whether a specific database or search strategy was utilized. A transparent description of the selection process would enhance reproducibility for other researchers.
2. To enhance the article, a brief methodology on verifying the applicable version, status, and effective date of each primary instrument is needed. A table or footnote listing the instrument, issuing body, issue date, effective date, and official source would be beneficial, particularly as the article differentiates current regulations from those effective in July 2027. Without this verification protocol, readers may struggle to confirm the accuracy of the authors’ claims regarding the status of various instruments.
3. The article critiques its focus on doctrinal analysis, highlighting the absence of empirical data. While it cites CBUAE’s 2024 insurance statistics, it calls for a better examination of the practical effects of reforms on the UAE's takaful market. Notably, it points out the lack of insights into market behaviour changes, such as entry rates and product innovation. The authors assert that reforms could lower legal due-diligence costs and enhance product administrability, yet these claims remain untested. Recognizing this limitation and suggesting future research directions could greatly improve the article.
4. Insufficient detail about the Higher Shari’ah Authority (HSA) in the UAE’s Shari'ah governance is presented. The article notes the HSA’s role as a benchmark and in resolving institutional disagreements but fails to explain member appointments, the resolution issuance process, and how conflicts with Internal Shari'ah Supervision Committee (ISSC) determinations are handled. More details would aid readers unfamiliar with these processes.
5. The article examines regulatory frameworks concerning Shari’ah compliance, focusing on policyholders' rights to sue for damages or rescind contracts. While it highlights that legislation does not void contracts over Shari’ah breaches, it lacks detail on practical remedies for policyholders, despite emphasizing the importance of participant protection.
6. Cross-border analysis is superficial, offering insights into choice of law and enforcement but lacking depth on foreign court rules like mandatory insurance and consumer protection in relation to UAE governing-law clauses. It questions scenarios where foreign courts might reject UAE clauses in takaful disputes, suggesting that a comprehensive exploration of these issues would improve the discussion.
7. To reconcile the Dana Gas citations, clarify the intended citation: (2017) EWHC 2928 (Comm) or (2018) EWHC 278 (Comm), ensuring consistent use. If both are relevant, detail their distinct procedural or substantive significance. For Beximco, include the neutral citation and rely on an authoritative report instead of a case aggregator for accuracy.

**Minor points **

1. Primary-law references and terminology. Add a complete, separate bibliography entry for the current CBUAE Regulation Regarding Takaful Insurance, including its official Rulebook source and relevant date information. Please also standardise the names “UAE Legislation/UAE Legislations” and “Shari’ah/Shariah” throughout.
2. Precision is required for two formulations: the term “proprietary and accounting-based” should be qualified to reflect that it arises from statutory and regulatory allocations, which do not address all insolvency or third-party proprietary issues. Furthermore, the reference to the company as the “sole usable party” is limited to beneficiary entitlements under the takaful policy and relevant regulations, rather than encompassing all claims related to its broader operations.
3. Copyedited the keyword “Takafu” to “Takaful.” Conducted a final check of punctuation around parenthetical citations and provided article-level references to facilitate easier verification of repeated legal propositions.

**Conclusion**

 Overall, the article makes a significant contribution to understanding the UAE’s takaful framework, showcasing strong legal analysis and a clear framework. While it provides balanced conclusions and practical implications, it lacks detail on legal material analysis, impacting transparency. Recommendations for minor revisions aim to enhance methodological and empirical aspects.

Is the work clearly and accurately presented and does it cite the current literature?

Yes

If applicable, is the statistical analysis and its interpretation appropriate?

Not applicable

Are all the source data underlying the results available to ensure full reproducibility?

Yes

Is the study design appropriate and is the work technically sound?

Yes

Are the conclusions drawn adequately supported by the results?

Yes

Are sufficient details of methods and analysis provided to allow replication by others?

Partly

Reviewer Expertise:

Islamic Banking and Finance, Research Methodologies, and Organisation Behaviour

I confirm that I have read this submission and believe that I have an appropriate level of expertise to confirm that it is of an acceptable scientific standard, however I have significant reservations, as outlined above.

# Response

**Published:** 2026-10-26

**Authors:** Moatasem al-Gheriani

We thank both reviewers for their careful and constructive assessments. We have revised the manuscript point by point while preserving its thesis, analytical framework, structure, and overall conclusion. All substantive revisions are visible in the accompanying tracked-changes manuscript. The principal changes are the addition of a transparent source-verification protocol and instrument register; expansion of the methodological limitations, HSA governance, participant remedies, and cross-border analysis; correction of the Dana Gas and Beximco authorities; qualification of the proprietary-separation and sole-suable-party formulations; and a final terminology, citation, and punctuation review.

 A temporal inconsistency became apparent during verification: the current CBUAE Regulation Regarding Takaful Insurance took effect on 14 September 2026, after the manuscript’s original cut-off date of 4 August 2026. The legal cut-off has therefore been updated to 26 October 2026. This allows the article to analyse the current regulation accurately while continuing to distinguish the standards displayed as effective from 15 July 2027 and their express preparatory compliance requirements.

**Strengths identified by the reviewer**

 We appreciate the reviewer’s positive assessment of the article’s timeliness and originality, its five-part analytical framework, balanced conclusion, practical drafting implications, comparative engagement, and implementation priorities. These strengths confirm the value of retaining the article’s central thesis and structure. No substantive response was required to the six positive observations, and the revisions below have been designed to strengthen the article without altering those features.

 Comment 1

**Reviewer comment**

*The methodology should explain the systematic selection and analysis of legal materials, including selection criteria, databases, and search strategy.*

**Author response**

 We agree. The revised methodology identifies the UAE Legislation portal and CBUAE Rulebook as the principal official databases, gives the instrument-number, title, and subject terms used to locate materials, and states the purposive selection criteria. Materials were included where they directly governed one of the five dimensions of legal certainty or established the relevant predecessor or transitional regime. Later provisions were compared with predecessors and cross-references, and foreign judgments were selected only to test governing-law and document-specific enforceability propositions.

**Change in manuscript**

 Section 2, Research methodology: new paragraph beginning “For this revision, the UAE Legislation portal and CBUAE Rulebook were searched…”

 Comment 2

**Reviewer comment**

*Add a method for verifying the version, status, and effective date of each primary instrument.*

**Author response**

 We agree and have implemented this request together with Reviewer 1’s first point. The revised methodology distinguishes issue, publication, and commencement dates, explains the hierarchy between formally issued instruments and a conflicting Rulebook display, and records the status of each principal instrument in a verification register.

**Change in manuscript**

 Section 2 and Table 1. Section 4.1 applies the protocol to the current 2026 regulation and the two standards displayed as effective from 15 July 2027.

 Comment 3

**Reviewer comment**

*Acknowledge the lack of empirical evidence and propose clearer future research on market behaviour, entry rates, product innovation, transaction costs, and participant confidence.*

**Author response**

 We agree. The manuscript now states more explicitly that the 2024 market figures are pre-reform context rather than an outcome measure, and that the study neither measures market entry or product innovation nor estimates effects on transaction costs or participant confidence. The future-research paragraph now identifies measurable outcomes and relevant confounders.

**Change in manuscript**

 End of Section 2: strengthened empirical limitation. Section 8: future research should track new licences and market entry, product launches, documented compliance costs, complaint or claims outcomes, and distinguish reform effects from premium growth, inflation, and other market changes.

 Comment 4

**Reviewer comment**

*Explain HSA member appointments, the issuance of resolutions, and the treatment of conflicts with ISSC determinations.*

**Author response**

 We agree. The revised discussion explains that Article 24(1)–(3) provides for five to seven HSA members with relevant expertise, appointed by CBUAE decision under a Board-approved charter for renewable three-year terms. It also explains the binding force of HSA resolutions and fatwas, the use of formal CBUAE notices to communicate adopted resolutions, the HSA approval required for ISSC appointments and dismissals, and the referral of disagreements among ISSC members or between the ISSC and the company’s board to the HSA for a binding and final opinion. We do not infer unverified internal voting or quorum procedures for the HSA.

**Change in manuscript**

 Section 4.2, new paragraph after the discussion of Article 24. Authorities added: Federal Decree-Law No. 6 of 2025, Articles 24 and 75, and HSA Resolution No. 18/3/2018.

 Comment 5

**Reviewer comment**

*Provide more detail on practical remedies available to policyholders or beneficiaries when Shari’ah compliance obligations are breached.*

**Author response**

 We agree. The revision separates three routes that should not be conflated. First, a beneficiary may pursue the company for an unpaid policy benefit under Articles 6.2–6.5 of the current regulation, with payment from fund assets. Second, a breach of a specifically incorporated contractual compliance undertaking may support performance or rescission under Article 234 of the Civil Transactions Law, subject to notice, materiality, cure, causation, and judicial assessment; compensation may be available where justified. Third, Article 148(3)–(8) of the Central Bank Law provides the complaint and insurance-dispute committee route and makes prior presentation to the committee a condition of judicial admissibility. Regulatory sanctions remain distinct from private remedies.

**Change in manuscript**

 Section 4.3, new paragraph beginning “In practical terms, a beneficiary seeking an unpaid policy benefit…” Article-level references have been added to the current takaful regulation, Civil Transactions Law, and Central Bank Law.

 Comment 6

**Reviewer comment**

*Deepen the cross-border analysis by explaining how mandatory insurance and consumer-protection rules may affect a UAE governing-law clause.*

**Author response**

 We agree. The revision adds a concrete EU illustration under the Rome I Regulation. It explains the restricted choices for non-large-risk insurance under Article 7(3), the preservation of compulsory-insurance requirements under Article 7(4), the non-derogable consumer protections potentially preserved by Article 6 subject to Article 7, and the distinct operation of overriding mandatory rules and public policy under Articles 9 and 21. The paragraph makes clear that the result depends on the insured risk, policyholder, and forum and does not establish a general refusal to apply UAE law.

**Change in manuscript**

 Section 7.2, new paragraph after the general limits on foreign enforcement. A full reference to Regulation (EC) No. 593/2008 on the law applicable to contractual obligations (Rome I) has been added.

 Comment 7

**Reviewer comment**

*Reconcile Dana Gas and improve the Beximco authority.*

**Author response**

 We agree. This point has been addressed as explained in the response to Reviewer 1, Comment 3. Dana Gas is now cited consistently to the 2017 preliminary-issue judgment, and Beximco now includes the neutral citation, authoritative report, and paragraph references.

**Change in manuscript**

 Sections 7.1 and 7.2 and the reference list.

 Minor points

 Comment 1

**Reviewer comment**

*Add the current CBUAE takaful regulation as a separate primary-law reference and standardise terminology.*

**Author response**

 Implemented. A separate complete entry for the current regulation has been added. “UAE Legislation” and “Shari’ah” are now standardised in the authors’ prose, subject to accurate reproduction of published titles.

**Change in manuscript**

 Reference list and full manuscript.

 Comment 2

**Reviewer comment**

*Qualify the proprietary-separation and sole-suable-party formulations.*

**Author response**

 Implemented. The proprietary formulation is tied to the statutory and regulatory allocation and expressly leaves insolvency and third-party questions open. The sole-suable-party statement is confined to beneficiary entitlements arising under the takaful policy.

**Change in manuscript**

 Abstract, Sections 1, 5.1, 5.2, and 9.

 Comment 3

**Reviewer comment**

*Correct the keyword and complete citation-level copyediting.*

**Author response**

 Implemented. “Takafu” has been corrected to “Takaful,” punctuation around parenthetical citations has been reviewed, and article-level references have been added throughout.

**Change in manuscript**

 Keywords, full manuscript, and reference list.

 Closing statement

 We respectfully submit that the revised manuscript addresses all requested revisions. The changes improve transparency, temporal accuracy, doctrinal precision, and verifiability while preserving the article’s thesis that the UAE reforms produce a layered and increasingly coherent takaful architecture, with remaining limits in private-law remedies, insolvency treatment, and cross-border enforcement.

# Reviewer report: Reviewer response for version 1

**DOI:** 10.5256/f1000research.210004.r523905

**Published:** 2026-09-30

**Authors:** Arief Fahmi Lubis

**Affiliations:** 1 Sekolah Tinggi Hukum Militer, Jakarta, Indonesia

Required minor revisions

1. Source-verification protocol. Add a short explanation in the methodology of how the authors verified the applicable version, status, and effective date of each primary instrument. A compact table or footnote identifying the instrument, issuing body, issue date, effective date, and official source would suffice. This is especially useful because the article distinguishes current regulation from standards scheduled to take effect in July 2027.
2. Primary-law references and terminology. Add a complete, separate bibliography entry for the current CBUAE Regulation Regarding Takaful Insurance, including its official Rulebook source and relevant date information. Please also standardise the names "UAE Legislation/UAE Legislations" and "Shari'ah/Shariah" throughout.
3. Dana Gas and Beximco citations. Reconcile the Dana Gas citations: the text refers to [2017] EWHC 2928 (Comm), whereas the reference list identifies [2018] EWHC 278 (Comm). If both decisions are intended, explain their distinct procedural or substantive relevance; otherwise use one correct citation consistently. The Beximco reference should preferably include the neutral citation and an authoritative report rather than relying principally on a case aggregator.
4. Precision of two formulations. Slightly qualify the statement that the separation is "proprietary and accounting-based" to make clear that it follows from the statutory and regulatory allocation and does not predetermine all insolvency or third-party proprietary questions. Also clarify that the company being the "sole suable party" concerns beneficiary entitlements under the takaful policy under the relevant regulation, rather than necessarily every conceivable claim connected with the wider operation.
5. Copyediting. Correct the keyword "Takafu" to "Takaful". Please also conduct a final check of punctuation around parenthetical citations and provide article-level references where repeated legal propositions would benefit from easier verification.
  These revisions are limited and do not require a change to the article's thesis, structure, or overall conclusion.

Is the work clearly and accurately presented and does it cite the current literature?

Yes

If applicable, is the statistical analysis and its interpretation appropriate?

Not applicable

Are all the source data underlying the results available to ensure full reproducibility?

No source data required

Is the study design appropriate and is the work technically sound?

Yes

Are the conclusions drawn adequately supported by the results?

Yes

Are sufficient details of methods and analysis provided to allow replication by others?

Partly

Reviewer Expertise:

Legal Studies

I confirm that I have read this submission and believe that I have an appropriate level of expertise to confirm that it is of an acceptable scientific standard.

# Response

**Published:** 2026-10-26

**Authors:** Moatasem al-Gheriani

General response

 We thank both reviewers for their careful and constructive assessments. We have revised the manuscript point by point while preserving its thesis, analytical framework, structure, and overall conclusion. All substantive revisions are visible in the accompanying tracked-changes manuscript. The principal changes are the addition of a transparent source-verification protocol and instrument register; expansion of the methodological limitations, HSA governance, participant remedies, and cross-border analysis; correction of the Dana Gas and Beximco authorities; qualification of the proprietary-separation and sole-suable-party formulations; and a final terminology, citation, and punctuation review.

 A temporal inconsistency became apparent during verification: the current CBUAE Regulation Regarding Takaful Insurance took effect on 14 September 2026, after the manuscript’s original cut-off date of 4 August 2026. The legal cut-off has therefore been updated to 26 October 2026. This allows the article to analyse the current regulation accurately while continuing to distinguish the standards displayed as effective from 15 July 2027 and their express preparatory compliance requirements.

 Reviewer 1  Arief Fahmi Lubis

*Report dated 30 September 2026  Recommendation  Approved with required minor revisions*

 Comment 1

**Reviewer comment**

*Add a source-verification protocol and a compact table or footnote identifying each primary instrument, issuing body, issue date, effective date, status, and official source, especially to distinguish current regulation from standards scheduled for July 2027.*

**Author response**

 We agree. The methodology now explains how the authors searched the UAE Legislation portal and CBUAE Rulebook, selected materials, cross-checked official texts and portal metadata, and distinguished issue, publication, and commencement dates. We also explain how amendment, repeal, saving, and transitional provisions were verified. A new verification table records the principal instruments, issuing bodies, dates, status, and official sources. Where an exact issue date is not displayed in the consulted official record, the table states this expressly rather than inferring a date.

**Change in manuscript**

 Section 2, Research methodology: two new methodological paragraphs and new Table 1, “Verification register of the principal UAE instruments.” The legal cut-off is updated from 4 August 2026 to 26 October 2026. Section 4.1 also clarifies that the current regulation became effective on 14 September 2026 and that the 2027 standards contain preparatory compliance-plan requirements in addition to their displayed effective date.

 Comment 2

**Reviewer comment**

*Add a separate bibliography entry for the current CBUAE Regulation Regarding Takaful Insurance and standardise “UAE Legislation/UAE Legislations” and “Shari’ah/Shariah.”*

**Author response**

 We agree. A standalone bibliography entry has been added for the current CBUAE Regulation Regarding Takaful Insurance, including the issuing body, effective date, status, supersession of the 2022 regulation, commencement provision, official Rulebook source, and verification date. “UAE Legislation” and “Shari’ah” are now used consistently in the authors’ prose. Original spellings are retained only where necessary to reproduce an official or published title accurately.

**Change in manuscript**

 References: new entry “CBUAE Rulebook: Regulation Regarding Takaful Insurance. 2026a.” Terminology has been standardised throughout the abstract, main text, references, and keywords.

 Comment 3

**Reviewer comment**

*Reconcile the Dana Gas citations and replace the Beximco case-aggregator reference with the neutral citation and an authoritative report.*

**Author response**

 We agree. The article now relies consistently on Dana Gas PJSC v Dana Gas Sukuk Ltd and others [2017] EWHC 2928 (Comm), the judgment on the preliminary issue that supports the proposition discussed in the text. The former [2018] EWHC 278 (Comm) bibliography entry concerned a later procedural application and has been removed because it is unnecessary for the present argument. The Beximco entry now includes [2004] EWCA Civ 19 and [2004] 1 WLR 1784, with paragraph references and a judgment source, instead of relying on CaseMine.

**Change in manuscript**

 Section 7.1: Beximco citation corrected and pinpointed to paragraphs 48–54. Section 7.2: Dana Gas analysis restated as an English judgment on the preliminary issue, with pinpoint references to paragraphs 42–47, 50–57, and 86. References: CaseMine and the 2018 Dana Gas entry replaced by full case references.

 Comment 4

**Reviewer comment**

*Qualify “proprietary and accounting-based” and limit the “sole suable party” proposition to beneficiary entitlements under the takaful policy.*

**Author response**

 We agree. The first formulation now links separation expressly to the statutory and regulatory allocation of contributions, assets, and liabilities. It also states that this allocation does not predetermine every insolvency-priority or third-party proprietary question. The second formulation now makes clear that the sole-suable-party rule concerns beneficiary entitlements arising under the takaful policy and does not make the company the only possible defendant for every tort, investment, corporate, or other claim connected with the wider operation.

**Change in manuscript**

 Sections 5.1 and 5.2, with corresponding qualifications also added to the abstract, introduction, and conclusion. Article-level references to the current regulation, especially Articles 4(h), 6.2–6.5, and 18.4, have been supplied.

 Comment 5

**Reviewer comment**

*Correct “Takafu” to “Takaful,” check punctuation around parenthetical citations, and provide article-level references for repeated legal propositions.*

**Author response**

 We agree. The keyword has been corrected. Parenthetical citations were moved before sentence-ending punctuation where required, inconsistent spaces were removed, and repeated propositions concerning the fund, operational model, claims, qard hasan, solvency, governance, and remedies now carry article-level references.

**Change in manuscript**

 Keywords and the full manuscript. Examples include current-regulation references to Articles 4, 5.2, 6.1–6.5, 8–14, 18.4, 21, 24, 27, and 29; Central Bank Law references to Articles 24, 75, 77, 105, 148, and 183–186; and Civil Transactions Law references to Articles 19, 234, 953, 955, and 967.

 We respectfully submit that the revised manuscript addresses all requested revisions. The changes improve transparency, temporal accuracy, doctrinal precision, and verifiability while preserving the article’s thesis that the UAE reforms produce a layered and increasingly coherent takaful architecture, with remaining limits in private-law remedies, insolvency treatment, and cross-border enforcement.
